# Mental illness and well-being: the central importance of positive psychology and recovery approaches

**DOI:** 10.1186/1472-6963-10-26

**Published:** 2010-01-26

**Authors:** Mike Slade

**Affiliations:** 1 Health Service and Population Research Department (Box P029), Institute of Psychiatry, King's College London, Denmark Hill, London, SE5 8AF UK

## Abstract

**Background:**

A new evidence base is emerging, which focuses on well-being. This makes it possible for health services to orientate around promoting well-being as well as treating illness, and so to make a reality of the long-standing rhetoric that health is more than the absence of illness. The aim of this paper is to support the re-orientation of health services around promoting well-being. Mental health services are used as an example to illustrate the new knowledge skills which will be needed by health professionals.

**Discussion:**

New forms of evidence give a triangulated understanding about the promotion of well-being in mental health services. The academic discipline of positive psychology is developing evidence-based interventions to improve well-being. This complements the results emerging from synthesising narratives about recovery from mental illness, which provide ecologically valid insights into the processes by which people experiencing mental illness can develop a purposeful and meaningful life. The implications for health professionals are explored. In relation to working with individuals, more emphasis on the person's own goals and strengths will be needed, with integration of interventions which promote well-being into routine clinical practice. In addition, a more societally-focussed role for professionals is envisaged, in which a central part of the job is to influence local and national policies and practices that impact on well-being.

**Summary:**

If health services are to give primacy to increasing well-being, rather than to treating illness, then health workers need new approaches to working with individuals. For mental health services, this will involve the incorporation of emerging knowledge from recovery and from positive psychology into education and training for all mental health professionals, and changes to some long-established working practices.

## Background

The World Health Organisation (WHO) declares that health is "*A state of complete physical, mental and social well-being and not merely the absence of disease or infirmity *"[1]. However, creating health-oriented rather than illness-oriented services has proved rather more difficult than the clarity of this declaration would suggest. Efforts to generate a science of illness have been very successful, with shared taxonomies to identify types of illness, established and validated interventions to treat and manage these identified illnesses, and clinical guidelines and quality standards available to increase efficiency and equity. These successes have not been mirrored by equivalent advances in applying the science of well-being within health services. The typical health worker will know a lot about treating illness, and far less about promoting well-being.

In this article we use mental health services as an exemplar of the issue, and explore how mental health services could more effectively promote well-being. Our central argument is that mental health workers will need new approaches to assessment and treatment if the goal is promoting well-being rather than treating illness. Well-being is becoming a central focus of international policy, *e.g*. Canada [2] and the United Kingdom [3]. In the same way that tertiary prevention is an important health promotion strategy, well-being is possible for people experiencing mental illness.

We will discuss two new emerging areas of knowledge which are highly complementary, and provide a counter-balance to the traditional focus of mental health services on deficit amelioration. We will identify how they link (and differ), and then explore their implications for mental health workers. Specifically, we will argue that assessment and treatment of the individual will need to change if the goal is promoting well-being rather than treating illness, and that there are also broader challenges for mental health professionals to become more outward-looking in their view of their role, and to construct their job as more than working with individuals. We will conclude that a focus on improving social inclusion, becoming social activists who challenge stigma and discrimination, and promoting societal well-being may need to become the norm rather than the exception for mental health professionals in the 21^st ^Century.

## Discussion

The WHO declaration about mental health is also clear: it is "*a state of well-being in which the individual realizes his or her own abilities, can cope with the normal stresses of life, can work productively and fruitfully, and is able to make a contribution to his or her community *" [1]. A relative lack of workforce skills in promoting well-being is particularly important in mental health services, since mental disorders directly impact on personal identity and ability to maintain social roles.

This distinction between mental illness and mental health is empirically validated, with only modest correlations between measures of depression and measures of psychological well-being, ranging from -0.40 to -0.55 [4,5]. A more statistically robust approach is a confirmatory factor model, which showed that the latent factors of mental health and mental illness in a US sample (n = 3,032) correlated at 0.53, indicating that only one quarter of the variance between measures of mental illness and mental health is shared [6].

Why is this distinction important? Because it points to the need for mental health professionals to support both the reduction of mental illness and the improvement of mental health. This will involve the development of further skills in the workforce. These skills will be based on two new areas of knowledge, each of which have emerged as distinct scientific areas of enquiry only in the past two decades.

### New area of knowledge 1: Recovery

People personally affected by mental illness have become increasingly vocal in communicating both what their life is like with the mental illness and what helps in moving beyond the role of a patient with mental illness. Early accounts were written by individual pioneers [7-12]. These brave, and sometimes oppositional and challenging, voices provide ecologically valid pointers to what recovery looks and feels like from the inside. Once individual stories were more visible, compilations and syntheses of these accounts began to emerge from around the (especially Anglophone) world, *e.g*. from Australia [13], New Zealand [14-17], Scotland [18,19], the USA [12,20,21] and England [22,23]. The understanding of recovery which has emerged from these accounts emphasises the centrality of hope, identity, meaning and personal responsibility [13,24,25]. We will refer to this consumer-based understanding of recovery as **personal recovery**, to reflect its individually defined and experienced nature [26]. This contrasts with traditional clinical imperatives - which we will refer to as **clinical recovery**- which emphasise the invariant importance of symptomatology, social functioning, relapse prevention and risk management. To note, this distinction has been referred to by other writers as recovery "from" versus recovery "in" [27]; clinical recovery versus social recovery [28]; scientific versus consumer models of recovery [29]; and service-based recovery versus user-based recovery [30].

Opinions in the consumer literature about recovery are wide-ranging, and cannot be uniformly characterised. This multiplicity of perspectives in itself has a lesson for mental health services - no one approach works for, or 'fits', everyone. There is no right way for a person to recover. Eliciting idiographic knowledge - understanding of subjective phenomema - is an important clinical skill. Nonetheless, some themes emerge. A first clear point of divergence from the clinical perspective is that recovery is seen as a journey into life, not an outcome to be arrived at: "*recovery is not about 'getting rid' of problems. It is about seeing people beyond their problems - their abilities, possibilities, interests and dreams - and recovering the social roles and relationships that give life value and meaning *" [31].

Many definitions of recovery have been proposed by those who are experiencing it [8,18]. We will use the most widely-cited definition that "*recovery is a deeply personal, unique process of changing one's attitudes, values, feelings, goals, skills, and/or roles. It is a way of living a satisfying, hopeful, and contributing life even within the limitations caused by illness. Recovery involves the development of new meaning and purpose in one's life as one grows beyond the catastrophic effects of mental illness *" [32]. It is consistent with the less widely-cited but more succinct definition that recovery involves "*the establishment of a fulfilling, meaningful life and a positive sense of identity founded on hopefulness and self determination *" [13].

One implication of these definitions is that personal recovery is an individual process. Just as there is no one right way to do or experience recovery, so also what helps an individual at one time in their life may not help them at another. If mental health services are to be focussed on promoting personal recovery, then this means there cannot be a single recovery model for services. This is a profound point, and challenging to established concepts such as clinical guidelines, evidence-based practice and care pathways. A recurring feature in recovery narratives is the individual engaging or re-engaging in their life, on the basis of their own goals and strengths, and finding meaning and purpose through constructing or reclaiming a valued identity and social roles. All of this points to well-being rather than treatment of illness. There is now a scientific discipline - positive psychology - devoted to the promotion of well-being.

### New area of knowledge 2: Positive Psychology

Positive psychology is the science of what is needed for a good life. This is not a new focus - proposing qualities needed for a good life is an activity dating back to Aristotle's investigation of *eudaimonia*, and builds on seminal work in the last Century by Antonovsky [33], Rogers [34] and Maslow [35]. But the emergence of a scientific discipline in this area is a modern phenomenon. Martin Seligman, often identified along with Mihaly Csikszentmihalyi as the founders of the discipline, suggests a definition [36]:

*The field of positive psychology at the subjective level is about valued subjective experiences: well-being, contentment, and satisfaction (in the past); hope and optimism (for the future); and flow and happiness (in the present)*.

*At the individual level, it is about positive individual traits: the capacity for love and vocation, courage, interpersonal skill, aesthetic sensibility, perseverance, forgiveness, originality, future mindedness, spirituality, high talent, and wisdom*.

*At the group level, it is about the civic virtues and the institutions that move individuals toward better citizenship: responsibility, nurturance, altruism, civility, moderation, tolerance, and work ethic*.

Research centres are developing internationally (e.g. http://positivepsychologyaustralia.org, http://cappeu.com, http://centreforconfidence.co.uk). Academic compilations of the emerging empirical evidence [37,38] and accessible introductions to the theory [39,40] and its applications [41] are becoming available. Findings from positive psychology are important to mental health services because its focus on a good life is as relevant to people with mental illness as to people without mental illness.

**Figure 1:**
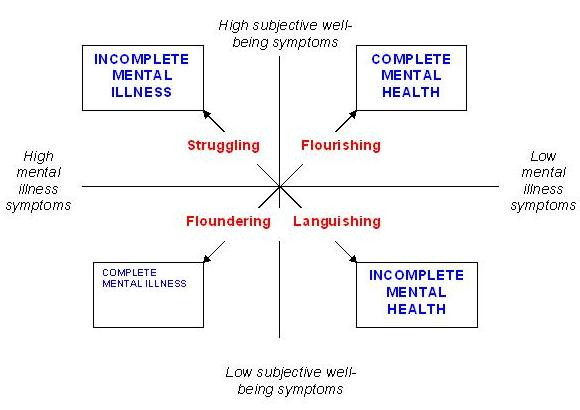
**Complete State Model of Mental Health**.

One key advance is in relation to empirical investigation of mental health. A conceptual framework is provided by the Complete State Model of Mental Health [42], proposed by Corey Keyes, and shown in Figure 1.

This model identifies two dimensions. Mental illness lies on a spectrum, from absent to present. Well-being also lies on a spectrum, from low to high.

This conceptual framework easily maps on to the themes emerging in the recovery literature. A perennial question about recovery is "How can you be recovered if you still have the mental illness?". Whatever answers are given, they can be only partial answers since the term recovery is an illness term. By contrast, access to mental health is open to all. This provides an alternative frame of understanding for recovery [26]:

**Personal recovery **involves working towards better mental health, regardless of the presence of mental illness

People with mental illness who are in recovery are those who are actively engaged in working away from Floundering (through hope-supporting relationships) and Languishing (by developing a positive identity), and towards Struggling (through Framing and self-managing the mental illness) and Flourishing (by developing valued social roles).

This concept of mental health has been operationalised into 13 dimensions, across the domains of emotional well-being, psychological well-being and social well-being [6,43]. These dimensions have been empirically validated [4,44], and are shown in Additional file 1.

Like mental illness, the concept of mental health can be expressed as a constellation of factors. Using the same diagnostic framework as DSM uses for major depression, the condition of Flourishing is defined as requiring high levels in Dimensions 1 (Positive affect) or 2 (Avowed quality of life) to be present, along with high levels on at least 6 of the 11 dimensions of positive functioning (Dimensions 3 to 13). Similarly, to be diagnosed as Languishing, individuals must exhibit low levels on one of the emotional well-being dimensions, and low levels on 6 of the remaining 11 dimensions. Adults who are neither flourishing nor languishing are said to be moderately mentally healthy. Finally, complete mental health is defined as the absence of mental illness and the presence of flourishing.

What is the prevalence of mental health, using these definitions? A cross-sectional assessment in the US population [43] (n = 3,032) is shown in Table 1.

**Table 1:** Prevalence of mental health and mental illness

Condition	Prevalence (%)
Mental Illness and Languishing	7
Mental Illness and Moderately Mentally Healthy	15
Mental Illness and Flourishing	1
Languishing (and no mental illness)	10
Moderate Mental Health (and no mental illness)	51
Complete Mental Health (Flourishing, no mental illness)	17

A similar US study of youth (n = 1,234) found 6% of 12 to 14 year olds Languishing, 45.2% with Moderate Mental Health, and 48.8% Flourishing, with respective proportions of 5.6%, 54.5% and 39.9% in 15 to 18 year olds [45].

These results have two profound implications. First, careful consideration should be given to the balance between research into mental illness and mental health. Among US adults with no mental illness, one in 10 are languishing and less than 2 in 10 are flourishing. The implicit expectation that research into mental illness will promote mental well-being is neither empirically justified nor a cost-free assumption - the opportunity costs for an illness-dominated research agenda may be high. For example, Flourishing is aligned with concepts such as self-righting, self-efficacy and mastery as characteristics which critically impact on the ability to self-manage. As Keyes puts it [6]:

*In particular, is languishing a diathesis for, and is flourishing a protective factor against, the onset and recurrence of mental illness? Conceptually, one can think of mental health as the continuum at the top of the cliff where most individuals reside. Flourishing individuals are at the healthiest and therefore farthest distance from the edge of this cliff; languishing places individuals very near the edge of the cliff. Hence, languishing may act as a diathesis that is activated by stressors that push individuals off the cliff and into mental illness*(p. 547)

There is empirical support for this proposition. One validated approach involves training for optimism, by modifying the three components of explanatory style (permanence, pervasiveness, personalisation) through transforming negative thinking into positive cognitive processes that promote flexible thoughts and resilience. A study involving 70 children at high risk of depression showed that this technique reduced depressive symptomatology and lowered incidence rates at 2-year follow-up [46]. In a mental health service context, there is also emerging evidence that positive life events are important protective factors [47]. A study of 260 people with severe mental illness showed that an increasing ability to engage in pleasurable activities leads to the ability to regulate depressive symptoms to the point where they did not impact on identity by eroding self-esteem [48].

The second implication is that it is possible to be moderately mentally healthy, or even flourishing, despite the presence of ongoing mental illness. In other words, personal recovery is possible even in the presence of current symptoms. Cook and Jonikas label this process as thriving, in which individuals rebuild lives with qualities better than before their difficulties began [49]. Interventions which support the individual in moving towards mental health may be as important as interventions which address the mental illness.

Positive psychology is specifically relevant to personal recovery. Factors identified by consumers as important for their recovery include hope, spirituality, empowerment, connection, purpose, self-identity, symptom management and stigma [30]. All but symptom management are almost entirely absent from professional training [50]. An influential framework, and one which could underpin the training of mental health professionals, is Seligman's theory of Authentic Happiness [51,52]. This identifies different types of good life:

1. The **Pleasant Life**, which consists in having as much positive emotion as possible and learning the skills to prolong and intensify pleasures

2. The **Engaged Life**, which consists in knowing your character (highest) strengths and recrafting your work, love, friendship, play and parenting to use them as much as possible

3. The **Meaningful Life**, which consists in using your character strengths to belong to and serve something that you believe is larger than just your self

4. The **Achieving Life**, which is a life dedicated to achieving for the sake of achievement.

This framework points to the possibility of different types of good life - which means that a range of approaches to promoting well-being are needed. For example, the positive psychology literature has addressed the question of how to lead an engaged life. A key emergent concept is flow, which requires two conditions [53]:

a) Perceived challenges that stretch (*i.e*. neither over-match nor under-utilise) existing skills - a sense that one is engaging challenges at the level of one's capacities

b) Clear proximal (short-term) goals and immediate feedback on progress

They define being **in flow **as:

*the subjective experience of engaging just-manageable challenges by tackling a series of goals, continuously processing feedback about progress, and adjusting action based on this feedback *(p. 90)

In terms of flow, a good life is one that involves complete absorption in what one does.

Flow is an important concept for mental health professionals to understand, since it is the structural opposite of positive emotion. Flow is a subjective experience, but unlike positive emotions it is not defined by feelings. Rather, it results from doing activities we like. Indeed, 80% of people report that when in flow, feelings and thinking are temporarily blocked [53]. This means that **feeling good is not always necessary for a good life**. Consequently, an automatic focus on taking away experiences of unhappiness (such as symptoms of depression) may be counter-productive. It is possible to experience authentic happiness by living a meaningful life that comes through full engagement. This of course has implications for how mental health services work - the aim may not be to help the person to feel better, but to re-engage in their life. What this means for mental health services is that a *central *challenge is supporting reasonable goal-setting and goal-striving. These goals need to be:

#### 1. Personally relevant, rather than meeting the needs of staff

There may of course be other reasons for staff-based care planning, but care plans focussed on clinical risk, medication compliance, relapse prevention and symptom reduction will not promote personal recovery

#### 2. The right level of challenge

The concept of a reasonable goal captures the balance in setting goals which are neither too easy (leading to boredom and distraction) nor too difficult (leading to anxiety and heightened self-awareness). A good life is not achieved by simply lowering expectations, as commentators from both left-wing politics (who want more justice) and right-wing politics (who want more excellence) have noted [54]. But nor is it achieved by raising expectations too high - recovery should be a journey, not a tread-mill.

#### 3. Proximal rather than distal

Short-term goals provide more opportunity to become engrossed in the experience, and make engaged goal-striving more likely

#### 4. Structured so that feedback is immediate and authentic

It is this immediate feedback loop that promotes full attentional awareness on the challenge

One approach to increasing well-being is therefore to support personally-relevant goal-setting and goal-striving activity. The Collaborative Recovery Model emphasises key recovery values of autonomy and self-determination [55], and builds on an established evidence base around personal goal-setting and goal-striving [56]. Preliminary evaluations of CRM are positive, showing improvements in staff attitudes (*e.g*. hopefulness) and knowledge [57]. A 10-site randomised controlled trial across three Australian states is underway.

Future-oriented goal-setting is not the only approach, and some traditions emphasise being over becoming: "*so we never live, but we hope to live; and as we are always preparing to be happy, it is inevitable we should never be so*" [58] (p. 87). A second approach to increasing well-being is to pay more attention to spiritual development and healing [59]: "*The healing process not only incorporates a new way of living with and controlling symptoms, but also an increasing adeptness of navigating social realms to overcome stigmatizing and discriminatory social-structural beliefs and practices. Re-authoring hinges on reclaiming a positive self-concept*." (p. 14). Healing as a spiritual activity [60], the role of moral experience [61], the role of community rather than individualism [62], the place of religion in mental health services [63] and approaches to supporting spirituality [64] are all contributors to well-being.

### Parallels between positive psychology and recovery

There are parallels between the position of recovery ideas in the mental health system and the position of positive psychology in the family of psychology disciplines [65]. Some points of convergence are shown in Additional File 2.

Two points of divergence can be identified [65]. First, the positive psychology focus has explicitly been on balancing the preoccupations of clinical psychology by distancing from the "*study of pathology, weakness, and damage*" [36]. Most empirical research has therefore involved people with either no mental illness or with mild to moderate common mental disorders such as depression and anxiety. An implicit, and sometimes explicit [66], dichotomous assumption is that healthy people will benefit from positive psychology, whereas people with mental illness will continue to require 'negative' psychology. There is no evidence for this assumption, and indeed the convergence of narratives from people with mental illness around key positive psychology themes (*e.g*. meaning, agency, empowerment, hope and resilience) suggests that the opposite may be true. As Resnick and Rosenheck put it [65]:

*Proponents of the recovery model would instead argue that the existence of "pathology" is not equivalent to "weakness and damage" and should not preclude a focus on what is healthy. The benefits of positive psychology might be even greater for people with severe psychiatric disabilities than for those without such impairments*. (p. 121)

A second point of divergence is methodological. Proponents of the recovery approach have focussed on developing position statements [67], consensus statements [68], frameworks [69], guidelines [70], and other action- and change-oriented approaches. This has been more successful at influencing policy than positive psychology. The relatively small amount of empirical recovery research has in general used inductive methods, such as collating and synthesising narratives. This is consistent with an emphasis on individual meaning and experience, since grouping the responses of participants necessarily reduces the granularity of analysis. However, the consequence is difficulty in making the intellectual case to clinicians with influence to change the mental health system, who tend to value nomothetic group-level evidence.

By contrast, positive psychology is unequivocally based on empirical research, and unlike recovery-focussed research has not avoided the use of nomothetic approaches, even to assess complex constructs such as meaning of life [71]. Indeed, it has been criticised for under-use of qualitative methods [72]. This scientific orientation has led to an emphasis on conceptual clarity, the use of scientific methods, and convergence on overarching theories [51]. The result is an academically credible scientific discipline [37], whose evidence is based on robust scientific methodologies [73]. It has not, however, yet been highly influential in international policy.

Why has there not been a greater rapprochement between these two, apparently compatible, groups? This may be because of their differing aims [65] - one is "*an intellectual movement, led by prominent academic psychologists, that challenges the dominance of "negative psychology"*, whereas the other is "*a grassroots movement of the disenfranchised that has placed itself apart from the human service professions, the academy, and the empirical research tradition*" (p. 121).

A second reason may be the name of the discipline. Positioning it as a branch of psychology invokes unhelpful tribal loyalties - it suggests a relevance to psychologists but not other types of researchers or professionals. The oppositional perspective of some positive psychologists reinforces this divergence [74]. The name is misleading - well-being is a potential focus for many disciplines. Anthropologists would help us understand the association between social systems and well-being. Geneticists will create designer-baby dilemmas when they can select embryos which are more likely to be happy. Sociology could investigate how the meaning of well-being is constructed and identify influences on its evolution. What are the neuroanatomical correlates of resilience? What philosophical perspective is associated with maximum well-being? Can we teach children compassion? The field is far larger than the name implies, and highly cross-disciplinary. A less loaded name for the discipline would be helpful, either a neologism (positology?) or a more neutral term such as positive well-being.

This divergence is impoverishing for both groups. The concordance between the fundamental aims of recovery in mental illness and of positive psychology suggests valuable lessons may be learned in both directions. For recovery, the development of a clinically credible evidence base, including randomised controlled trial evidence, has potential to be an important pathway to transforming mental health services. The preponderance of good ideas and relative paucity of evaluative research has been highlighted as a key problem in getting recovery-focussed practice into everyday mental health practice [75]. Whilst there are tensions between the values of evidence-based mental health and recovery [76], there is no fundamental incompatibility [77]. For example, the use of an invariant primary outcome for all participants in a clinical trial does not capture the individual nature of recovery, and innovative approaches to individualising clinical end-point measurement are now being evaluated in the REFOCUS Study http://researchintorecovery.com. Similarly, the need of professionals for a conceptual framework to understand recovery which does not become a 'model' showing the 'right' way to recovery is addressed in the Personal Recovery Framework which gives primacy to identity over illness [78].

For positive psychology, the incorporation of the central recovery focus on the individual and their differing ways of seeing the world (including giving primacy to familial or cultural affiliation over personal identity) will address criticisms that it is ethnocentric (being based mainly on US research) and overly concerned with the experience of individuals rather than groups [78,79]. Additionally, if there are ways in which people with mental illness are outliers (*e.g*. in having a relatively low ratio of protective to risk factors), then excluding them from consideration makes the development of generalisable theories more difficult.

### Implications for mental health assessment practices

How can a person with mental illness be assessed if the clinical goal is to promote well-being? Clinical assessment should focus on four dimensions [80]:

1. Deficiencies and undermining characteristics of the person

2. Strengths and assets of the person

3. Lacks and destructive factors in the environment

4. Resource and opportunities in the environment

Traditional clinical assessment practice - exemplified by the mental state assessment - focuses almost exclusively on dimension 1. This focus has arisen for several reasons. First, multi-dimensional assessment is hard work. Each dimension is dynamic and changing, and inter-dependent in complex ways. Holding this complexity is intellectually demanding, and requires a tentative stance and openness to changing understanding. It is much easier and in some ways more rewarding to be the clinical expert, who can summarise the problems of the person (*i.e*. dimension 1) with a pithy piece of professional language. This issue will reduce with the development of a shared taxonomy and language for dimensions 2 to 4. This is beginning to emerge. For example, the concept of character strengths has been disaggregated into six core virtues of wisdom, courage, humanity, justice, temperance and transcendence [81]. Similarly, positive affect has been disaggregated into Joviality (*e.g*. cheerful, happy, enthusiastic), Self-Assurance (*e.g*. confident, strong, daring) and Attentiveness (*e.g*. alert, concentrating, determined) [82].

Second, the expectation in the mental health system that it is the person who is going to be treated inevitably leads to a focus of attention on the individual. This of course is a consequence of clinical (and patient) beliefs about what the job is, and doesn't have to be the case.

Third, the clinician's illusion means that professionals don't see people as often when they are coping [83], so they gain the false impression they cannot cope or self-right.

Finally, the questions asked impose a structure on the dialogue, and influences content. The highly practised deficits-focussed discourse of taking a psychiatric history systematically identifies all the deficient, inexplicable, different and abnormal qualities and experiences of the person. This focus on deficits (and the other Ds: difficulties, disappointment, diagnosis, disease, disability, disempowerment, disenfranchisement, demoralisation, dysfunction) reinforces an illness identity, and the person disappears. Up close, nobody is normal: a deficit-focussed discourse will always elicit confirmatory evidence for an illness-saturated view of the person.

An alternative approach is possible [84]. In assessment, this involves a greater emphasis on the individual's goals and strengths, an approach which has been developed and evaluated in the Strengths Model [85]. Other approaches which emphasise well-being over deficits in assessment processes are person-centred planning [86,87] and Wellness Recovery Action Planning [88]. What these have in common is an assumption that it is more productive to focus on what the person wants in their life and what they can do towards their own goals than on what the professional thinks is in the person's best interests and on what the person cannot do.

### Interventions in mental health services to promote well-being

What interventions increase levels of well-being or amplify existing strengths?

### Cognitive behavioural therapy (CBT)

This psychological intervention will be familiar to most clinical readers, so no introduction will be given. Competently-provided CBT is aligned with many elements of promoting recovery and personal well-being: a focus on personally-valued rather than service-valued goals; responsibility for change lies with the patient not the therapist; the development of meta-cognitive awareness - an awareness of thoughts being distinct from self - which creates the context in which a positive identity can flourish, despite the presence of ongoing symptoms of mental illness; enhancing self-management skills and reinforcing interdependence and independence rather than dependence, leading to sustained gains after the end of the formal therapy; and an emphasis on homework, reality testing and learning opportunities which all contribute to keeping the person in their life during therapy. If unhappiness is caused by a mismatch between self and ideal-self images, then CBT has the potential to focus on the environmental reality as much as the personal interpretation of experience. This points to a wider role for professionals, a point we will return to. Recent approaches to CBT explicitly focus on building strengths and resilience [89].

### Mindfulness

Meditation is "*a family of techniques which have in common a conscious attempt to focus attention in a non-analytical way, and an attempt not to dwell on discursive, ruminative thought*" [90]. Teaching meditation to members of the public increases self-reported happiness and well-being, changes which are corroborated by healthier EEG readings, heart rates and flu immunity [91].

Meditation has been applied to mental health issues, such as anger [92] and - in the form of mindfulness-based cognitive therapy (MBCT) - depression [93]. Mindfulness, like prayer [94], is a form of meditation which involves attending non-judgmentally to all stimuli in the internal and external environment but to avoid getting caught up in (*i.e*. ruminating on) any particular stimulus. Mindfulness requires a different mind-set to the quick-fix of a magic pharmacological or psychological bullet. Just as becoming a top-class violinist requires 10,000 hours of practice with a competent teacher [95], so too mindfulness needs to become a way of life if it is to transform identity. It involves changing habits:

• enhancing meta-cognitive awareness by noticing what one is thinking about

• developing the ability to urge-surf by noticing but not being caught up in rising cognitions

• developing cognitive fluidity - taking habits from one space and using in another (*e.g*. using metaphors: thoughts as passing cars; thoughts as clouds; hare brain, tortoise mind)

• paying attention to a wider range of the available percept or experiences

The pay-off in terms of well-being is high. Mindfulness has the potential to lead to a reconstructed, more complex identity, in which self and thought are separated. Development of a watching self gives a different means of responding to and working on experiences of mental illness. Developing habits of greater occupation of the available attention reduces rumination and increases being in the moment - the flow concept we discussed earlier [96]:

*...by increasing the amount of time a person spends thinking grateful and calming thoughts, there is simply less time to think upsetting and ''unhelpful'' thoughts. Assuming that attention is a zero-sum game, the most efficient way to reduce negative and increase positive thoughts and emotions may be to focus on increasing the positive*.(p. 28)

Overall, the personal qualities cultivated through mindfulness practice are nonjudging, nonstriving, acceptance, patience, trust, openness, letting go, gentleness, generosity, empathy, gratitude and lovingkindness [97] - qualities which are highly relevant to the personal recovery journey of people with mental illness.

### Narrative psychology

A further clinical approach emerges from a sub-discipline called *narrative psychology*, which investigates the value of translating emotional experiences into words. This brings together insights from three strands of research (primarily from European and American cultures) [98]:

1. Inhibition - not talking about emotional trauma is unhealthy

2. Cognitive - development of a self-narrative allows closure

3. Social dynamics - keeping a secret detaches one from society.

One approach involves asking people to write about (or in other ways generate an account of) their experiences, as a means of making sense of their own story. The most beneficial story content includes placing the story in a context appropriate to its purpose, the transformation of a bad experience into a good outcome, and the imposition of a coherent structure [99]. Developing stories about growth, dealing with difficult life events and personal redemption all contribute to a positive narrative identity [100]. Empirical evidence suggests that this approach is particularly beneficial for groups who, as a whole, are not as open about their emotions: men [101], people with high hostility [102], and people with alexythimia [103].

### Positive Psychotherapy

An approach which brings together several of these methods is positive psychotherapy (PPT) [104]. The focus in PPT is on increasing positive emotion, engagement and meaning. For example, groups for depression undertake a series of weekly exercises. Week 1 (Using Your Strengths) involves using the Values in Action Inventory of Strengths [81] to assess your top five strengths, and think of ways to use those strengths more in your everyday life. Week 2 (Three Good Things/Blessings) involved writing down three good things every evening that happened today, and why you think they happened. Week 3 (Obituary/Biography) involves imagining that you have passed away after living a fruitful and satisfying life, and writing an essay summarising what you would most like to be remembered for. Week 4 (Gratitude Visit) involves thinking of someone to whom you are very grateful, but whom you have never properly thanked, composing a letter to them describing your gratitude, and reading it to the person by phone or in person. Week 5 (Active/Constructive Responding) involves reacting in a visibly positive and enthusiastic way to good news from someone else at least once a day. Week 6 (Savouring) involves once a day taking the time to enjoy something that you usually hurry through, writing write down what you did, how you did it differently, and how it felt compared to when you rush through it. These exercises are intended to amplify components of Authentic Happiness [51]. Randomised controlled trials of group PPT with mild to moderately depressed students (n = 40) and individual PPT with severely depressed mental health clients (n = 46) both showed gains in symptom reduction and happiness, with moderate to large effect sizes and improvement sustained at one-year follow-up [104].

We have considered some approaches to focussing more on strengths, goals and preferences. However, if mental health services are to fully support recovery and promote well-being, it may not be enough to simply counter-balance a focus on individual deficit with a focus on individual capability, since this leaves unchallenged the clinical belief that treatment is something you do first, after which the person gets on with their own life. This is highlighted as an unhelpful approach in the accounts from people who write about their recovery from mental illness. For example, Rachel Perkins notes [105]:

*Mental health problems are not a full time job - we have lives to lead. Any services, or treatments, or interventions, or supports must be judged in these terms - how much they allow us to lead the lives we wish to lead*.

### Societal implications

We therefore now raise some potential implications of positive psychology for the job of the mental health professional at the social, rather than individual, level. This is underpinned by an emerging understanding of the importance of relationships and connection for individual and social well-being. For example, an international consortium of 450 academics has recently produced reports about determinants and influences on well-being [106]. This important document has been summarised by the New Economics Foundation http://www.neweconomics.org as Five Ways To Wellbeing: Connect (to others, individually and in communities); Be active; Take notice (of the world); Keep learning; and Give (*e.g*. smile, volunteer, join in). It is no coincidence that these are all outward-looking recommendations, more about engaging in and living life to the full than sorting out any internal or intrapsychic disturbances. Stigma and discrimination stop people with mental illness from exercising their full rights as citizens and meeting their human needs for connection [107]. Therefore, the role of the mental health professional should be about challenging stigma and creating well-being-promoting societies as well as treating illness.

### Mental health professionals can improve social inclusion

Supporting people using mental health services from accessing normal citizenship entitlements is a central (*i.e*. not an optional extra) part of the job. We illustrate this in relation to employment.

If a single outcome measure had to be chosen to capture recovery, there would be a case to make that it should be employment status. Not because of a value about economic productivity, but because work has so many associated benefits. There is now a strong evidence base that Individual Placement and Support (IPS) approaches which support the person to find and maintain mainstream employment are better than training the person up in separate sheltered employment schemes in preparation for mainstream work [108,109]. Mental health professionals can increase the access of service users to the valued social role of work by supporting the development of employment schemes [110].

One specific work opportunity is within mental health services. These are often large employers - the National Health Service in the UK is the largest employer in Europe. However, health services have a history of poor recruitment and retention approaches to attracting people with declared mental illness to work for them [31]. (Of course, many people working in these services have an undisclosed history of mental illness.) This is a wasted opportunity, and reinforces stigmatising us-and-them beliefs in the work-force. Actively encouraging applications from people who have used mental health services for all posts, and positively discriminating between applicants with the same skill level in favour of people with a history of mental illness are two relevant approaches. They directly challenge "*the common tendency in human service organisations to see workers as either health and strong and the donors of care, or as weak and vulnerable recipients*" [108].

There are other ways in which mental health professionals and teams can improve social inclusion. A common experience of workers in the mental health system is frustration - a sense that these ideas about social inclusion, employment and social roles are all well and good, but impossible to implement within the existing constraints. But resources can become available by spending allocated money differently. This is the approach used by The Village http://www.mhavillage.org, a mental health service in inner-city Los Angeles working with homeless and severely mentally ill clients. The service decided to undergo a 'fiscal paradigm shift', by spending money to promote wellness and recovery (especially by creating pathways back into employment) rather than promote stability and maintenance. This involved transforming from being an organisation which spent most of its allocated money on acute hospitalisation (28%), long-term care (23%) and out-patient therapy (23%) to one spending on individualised case management (41%), work (25%) and community integration (12%) [111]. Hospitalisations and living in institutional residence are markedly reduced for members attending the Village [112], allowing the money saved to be re-invested in work-supporting services.

A further contribution from the clinician can be educating local employers about their legal duties under relevant discrimination legislation and about reasonable work-place adjustments for people with mental illness. The accommodations can relate to People (focussing on interpersonal challenges), Places (focussing on where the work takes place), Things (focussing on equipment needed to do the job) or Activities (focussing on the work tasks). For people with physical disability, accommodation needs tend to relate to Places and Things. This is what employers are used to. In mental illness, People issues are often the central issue. Employers need educating about how these interpersonal needs can be tended to, which might include [108]:

• addressing concentration problems by having a quieter work place with fewer distractions rather than an open-plan office

• the need to have some time away from other workers

• enhanced supervision to give feedback and guidance on job performance

• allowing the use of headphones to block out distracting noise (including hearing voices)

• flexibility in working hours, *e.g*. to attend clinical appointments or work when less impaired by medication

• mentor scheme for on-site orientation and support

• the need to talk to a supporter (*e.g*. a job coach) during a lunch break

• clear job description for people who find ambiguity and uncertainty difficult

• prior discussion about how leave due to illness will be managed, *e.g*. allowing the use of accrued paid and unpaid leave

• relocation of marginal job functions which are disturbing to the individual

Alongside this direct contribution to improving social inclusion, well-being focussed mental health professionals of the future will also have a contribution to make to policy.

### Mental health professionals can increase societal well-being

If a new knowledge base around well-being is integrated by mental health professionals into their practice, then this creates opportunities to influence social and political priorities. The position power and status of the role allow authoritative communication with the aim of influencing society and increasing wellbeing both for the general population and specifically for people with experience of mental illness. A few examples will illustrate this re-orientation.

Does money bring happiness? Above a certain level (estimated by Richard Layard as US$20,000pa [54]), the answer is no - relative wealth is more of an influence on happiness than absolute wealth [113]. A salary of $50,000 where average salaries are $25,000 is preferred to a salary of $100,000 where the average is $250,000 [114]. If social comparison influences well-being, what are the implications for policy? For example, do social structures such as gated communities and private schools harm us all? Contrary to intuition, those within the enclave aren't any happier because they are no wealthier than their comparison group, and those outside have a visibly wealthier reference group.

Television is a powerful influence, both because it encourages social comparison and because of its innate effects. Researchers have observed consistently adverse changes following the introduction of television into new communities. In Bhutan, this was followed by increased family break-up, crime and drug-taking, alongside reduced parent-child conversation [115]. In Canada, social life, participation in sports and level of creativity were all negatively impacted [116]. Homicide rates go up after televised heavyweight fights [117], and suicide rates increase after on-screen portrayals [118]. Television content leads to an inflated estimate of adultery and crime rates [119], and negative self-appraisal [120]. Given the average Briton watches 25 hours of television per week [121] - with similar levels in the US [54] - what does this imply for media regulation?

When making a social comparison, the reference group influences well-being: Olympic bronze medal winners (who compare themselves with people missing out on a medal) are happier than Silver medal winners (who compare themselves with the victor) [122]. For mental health, this may mean that anti-stigma campaigns focussed on promoting mental health literacy and identifying when to seek professional help actually increase negative social comparisons and reduce well-being. High-profile people talking about their own experiences are better at reducing the social distance and difference experienced by people with mental illness [107].

In contrast to salary, 4 weeks holiday when others have 8 weeks is preferred over 2 weeks when others have 1 week [114]. Would a national policy of compulsory flexible working arrangements (*e.g*. annualised hours) reduce work-related stress and consequent mental illness? More generally, the fact that people who win Oscars live longer than unsuccessful nominees [123] may point to the importance of achievement for longevity. If we want people to live longer, should we focus on developing community-level opportunities for participation, connection and mastery? Should services for particularly marginalised groups, such as people with mental illness, put some of their resources towards celebrating and amplifying success?

What are the sources of happiness? The Big Seven influences on happiness explain 80% of the variance in happiness: Family relationships, Financial situation, Work, Community and friends, Health, Personal freedom and Personal values [54]. The effects on happiness of problems in each domain have been estimated, on the basis of international surveys of factors associated with happiness [124,125]. Using a scale from 10 (no happiness) to 100 (total happiness), the fall in happiness associated with separation (compared with marriage) is 8 points, with unemployment or poor health is 6 points, with personal freedom is 5 points, with saying no to "God is important in my life" (personal values) is 3.5 points, with a national increase of 10% in unemployment is 3 points, and with a drop in family income by a third is 2 points.

Can these seven identified influences be used by mental health services to directly increase happiness, rather than continuing with attempts to reduce unhappiness? This will involve meeting three challenges. First, traditional professional training only focuses on one of these seven influences: health. Second, interventions to promote health which increase personal freedom and are concordant with personal values will increase happiness more than those which impinge on personal freedom or which deny or discount personal value. This will require clinical decision-making to focus as much on values and freedom as on intervention effectiveness - echoing the call for ethics before technology by Bracken and Thomas [126]. Third, most influences on happiness are social rather than intrapsychic, yet most mental health interventions are at the level of the individual. Overall, this is not to argue for more centralised control *per se*, but rather to highlight that this knowledge should be more visible in public debate, so that both social policy and individual choices are informed by our best scientific understanding of contributors to well-being.

We finish on an optimistic note. One reason for raising some of these implications is to highlight their relative absence from sociopolitical debate. Although there is good evidence that being happy and cheerful is associated with improved brain chemistry, blood pressure and heart rate [127,128], and with living longer [129], this kind of evidence does not yet feature prominently in public debate. If skilled professionals with an interest in promoting well-being don't point out that a high turnover of local residents create communities which are less cohesive [130] and more violent [131] then who will inject this information into social policy? This opens up innovative environmental approaches to fostering well-being, like the simple act of closing most points of entry to a housing estate which led to an increased sense of community and a 25% reduction in mental illness rates [132]. Similarly, the pernicious effects of a societal value that we *must *make the most of everything is becoming clear. People who constantly worry about missing opportunities - so-called hyper-optimisers - have more regrets, make more social comparisons and are less happy than people who are happy with what is good enough [133]. An empirically-informed policy-making approach would recognise the toxic consequences for well-being of societies which encourage unfavourable social comparison, continuous reoptimisation to make the best of every opportunity, and living for the future rather than savouring the present.

Research into mental illness proceeds apace. Advances in understanding are being generated by genetic, genomic, proteomic, psychological and epidemiological studies, among other disciplines. These advances are to be welcomed, and should continue to inform clinical practice. The challenge is to also integrate and apply the evidence base around well-being, so that mental *health *professionals of the future inform social policy as well as treating mental illness.

## Summary

• Two new sources of knowledge are now available to mental health professionals: collated syntheses of narratives of recovery from mental illness, and empirical evidence about well-being from the academic discipline of positive psychology

• These two sources are highly complementary, and provide a counter-balance to the traditional focus of mental health services on deficit amelioration

• Assessment and treatment of the individual will need to change if the goal is promoting well-being rather than treating illness

• There are also broader challenges for mental health professionals to become more outward-looking in their view of their role, and to construct their job as more than working with individuals

• A focus on improving social inclusion, becoming social activists who challenge stigma and discrimination, and promoting societal well-being may need to become the norm rather than the exception for mental health professionals in the 21^st ^Century.

## Competing interests

The author declares that they have no competing interests.

## Author's information

Dr Mike Slade is a Reader in Mental Health Services Research at the Institute of Psychiatry, and a consultant clinical psychologist in rehabilitation with South London and Maudsley Mental Health NHS Foundation Trust. Mike's main research interests are recovery-focussed and outcome-focussed mental health services, user involvement in and influence on mental health services, staff-patient agreement on need, and contributing to the development of clinically useable outcome measures, including the Camberwell Assessment of Need and the Threshold Assessment Grid. He has written over 120 academic articles and seven books, including Slade M (2009) *Personal recovery and mental illness*, Cambridge: Cambridge University Press. He is keen to disseminate an understanding of recovery to the field through free-to-download booklets, such as Shepherd G, Boardman J, Slade M (2008) *Making Recovery a Reality*, London: Sainsbury Centre for Mental Health (downloadable from http://www.scmh.org.uk) and Slade M (2009) *100 ways to support*recovery, London: Rethink (downloadable from http://www.rethink.org). He has acquired over £7 m of grant funding, including a £2 m NIHR Programme Grant for Applied Research for the five-year REFOCUS study to develop a recovery focus in adult mental services in England.

## Pre-publication history

The pre-publication history for this paper can be accessed here:

http://www.biomedcentral.com/1472-6963/10/26/prepub

## Supplementary Material

Additional file 1 **Operationalisation, definition and examples of three domains of mental health**. Table showing operationalisation, definition and examples of three domains of mental health. Click here for file

Additional file 2 **Points of convergence between recovery in mental illness and positive psychology**. Table showing points of convergence between recovery in mental illness and positive psychology. Click here for file
