# Supplementary material for: Mental illness and well-being: the central importance of positive psychology and recovery approaches
Source: BMC Health Serv Res. 2010 Jan 26;10:26. doi: 10.1186/1472-6963-10-26 (PMC2835700; doi:10.1186/1472-6963-10-26)
Supplement: Additional file 1 — Operationalisation, definition and examples of three domains of mental health. Table showing operationalisation, definition and examples of three domains of mental health. [file 1472-6963-10-26-S1.DOC]

**Table: Operationalisation, definition and examples of three domains of mental health**

| **Domain** | **Dimension** | **Definition** | **Example** |
| --- | --- | --- | --- |
|  |  |  |  |
| *Emotional*  *well-being* | 1. Positive affect | Regularly cheerful, interested in life, in good spirits, happy, calm and peaceful, full of life | *I feel happy and engaged in life most of the time* |
|  |  |  |  |
|  | 2. Avowed quality of life | Mostly or highly satisfied with life overall or in domains of life | *My life is good, and I wouldn’t change it* |
|  |  |  |  |
|  |  |  |  |
| *Psychological well-being* | 3. Self-acceptance | Holds positive attitudes toward self, acknowledges, likes most parts of self, personality | *When I look at the story of my life, I am pleased with how things have turned out so far* |
|  |  |  |  |
|  | 4. Personal growth | Seeks challenge, has insight into own potential, feels a sense of continued development | *For me, life has been a continuous process of learning, changing and growth* |
|  |  |  |  |
|  | 5. Purpose in life | Finds own life has a direction and meaning | *Some people wander aimlessly through life, but I am not one of them* |
|  |  |  |  |
|  | 6. Environmental  mastery | Exercises ability to select, manage, and mould personal environs to suit needs | *I am good at managing the responsibilities of daily life* |
|  |  |  |  |
|  | 7. Autonomy | Is guided by own, socially accepted, internal standards and values | *I have confidence in my own opinions, even if they differ from most other people* |
|  |  |  |  |
|  | 8. Positive relations with others | Has, or can form, warm, trusting personal relationships | *People would describe me as a giving person, willing to share my time with others* |
|  |  |  |  |
|  |  |  |  |
| *Social*  *well-being* | 9. Social acceptance | Holds positive attitudes toward, acknowledges, and is accepting of human differences | *I believe people are kind* |
|  |  |  |  |
|  | 10. Social actualization | Believes people, groups, and society have potential and can evolve or grow positively | *The world is becoming a better place for everyone* |
|  |  |  |  |
|  | 11. Social contribution | Sees own daily activities as useful to and valued by society and others | *I have something valuable to give to the world* |
|  |  |  |  |
|  | 12. Social coherence | Interested in society and social life and finds them meaningful and somewhat intelligible | *I find it easy to predict what will happen next in society* |
|  |  |  |  |
|  | 13. Social integration | A sense of belonging to, and comfort and support from, a community | *My community is a source of support* |
|  |  |  |  |
