# Supplementary material for: Mental illness and well-being: the central importance of positive psychology and recovery approaches
Source: BMC Health Serv Res. 2010 Jan 26;10:26. doi: 10.1186/1472-6963-10-26 (PMC2835700; doi:10.1186/1472-6963-10-26)
Supplement: Additional file 2 — Points of convergence between recovery in mental illness and positive psychology. Table showing points of convergence between recovery in mental illness and positive psychology. [file 1472-6963-10-26-S2.DOC]

**Table: Points of convergence between recovery in mental illness and positive psychology**

| **Point of convergence** | **Recovery example** | **Positive Psychology example** |
| --- | --- | --- |
|  |  |  |
| The system from which they emerge has been criticised for being overly focussed on deficits, problems, disorder and pathology | System = mental health services | System = other psychology specialties, especially Clinical |
|  |  |  |
| Primacy is given to lived experience | Recovery is a personal journey, which the individual experiences | The experience of a good life is subjective and idiosyncratic |
|  |  |  |
| The emphasis is on making life better, rather than making life less bad | Recovery is about a meaningful life – symptom reduction may not be necessary | Positive psychological interventions focus on increasing well-being, not reducing distress |
|  |  |  |
| The consequent tasks are active and positive, rather than avoidant and negative | Recovery involves raising hope, finding meaning, developing identity and taking responsibility | A good life is pleasant, engaged, meaningful or achieving |
|  |  |  |
| Success can only be judged by the individual | Consumer assessments are more meaningful than staff assessments | What constitutes a good life varies from person to person, and can only be judged by the individual |
|  |  |  |
